# Advances in implantable bionic devices for blindness: a review

**DOI:** 10.1111/ans.13616

**Published:** 2016-06-14

**Authors:** Philip M. Lewis, Lauren N. Ayton, Robyn H. Guymer, Arthur J. Lowery, Peter J. Blamey, Penelope J. Allen, Chi D. Luu, Jeffrey V. Rosenfeld

**Affiliations:** ^1^ Department of NeurosurgeryAlfred HospitalMelbourneVictoriaAustralia; ^2^ Department of Surgery, Central Clinical SchoolMonash UniversityMelbourneVictoriaAustralia; ^3^ Monash Vision Group, Faculty of EngineeringMonash UniversityMelbourneVictoriaAustralia; ^4^ Monash Institute of Medical EngineeringMonash UniversityMelbourneVictoriaAustralia; ^5^ Centre for Eye Research AustraliaThe Royal Victorian Eye and Ear HospitalMelbourneVictoriaAustralia; ^6^ Department of OphthalmologyThe University of MelbourneMelbourneVictoriaAustralia; ^7^ Department of SurgeryThe University of MelbourneMelbourneVictoriaAustralia; ^8^ Bionics Institute, Department of Medical BionicsThe University of MelbourneMelbourneVictoriaAustralia; ^9^ F. Edward Hébert School of MedicineUniformed Services University of the Health SciencesBethesdaMarylandUSA

**Keywords:** bionics, blindness, brain, prosthesis, retina, vision

## Abstract

Since the 1950s, vision researchers have been working towards the ambitious goal of restoring a functional level of vision to the blind via electrical stimulation of the visual pathways. Groups based in Australia, USA, Germany, France and Japan report progress in the translation of retinal visual prosthetics from the experimental to clinical domains, with two retinal visual prostheses having recently received regulatory approval for clinical use. Regulatory approval for cortical visual prostheses is yet to be obtained; however, several groups report plans to conduct clinical trials in the near future, building upon the seminal clinical studies of Brindley and Dobelle. In this review, we discuss the general principles of visual prostheses employing electrical stimulation of the visual pathways, focusing on the retina and visual cortex as the two most extensively studied stimulation sites. We also discuss the surgical and functional outcomes reported to date for retinal and cortical prostheses, concluding with a brief discussion of novel developments in this field and an outlook for the future.

## Introduction

Since the 1950s, vision researchers have been working towards the goal of restoring a functional level of vision to the blind. There are a variety of approaches to achieving this goal; however, a common technique is to electrically stimulate the visual pathways with a vision prosthesis, or ‘bionic eye’. The underlying premise of such stimulation is to evoke neuronal activity at a site within the visual pathway that remains functional irrespective of the underlying cause of blindness. This activity then propagates along the remaining intact visual pathway to the visual cortex, resulting in some form of visual perception.

Globally, between 32 and 39 million people suffer from blindness, with the most common and treatable causes being cataract or uncorrected refractive error.^1^ Blindness resulting from retinal disease, such as age‐related macular degeneration (AMD) and inherited dystrophies such as retinitis pigmentosa (RP), often leaves the inner retina and optic nerve relatively intact, rendering sufferers of these conditions candidates for a retinal vision prosthesis. Conversely, damage to the inner retina or optic nerve requires a prosthesis that stimulates more distal sites along the visual pathway.

With these factors in mind, the site targeted for stimulation will differ according to the extent and location of disease or damage. Those sites already identified as suitable include the retina, optic nerve, lateral geniculate nucleus and the visual cortex. The relative benefits and drawbacks of each are numerous and have been previously discussed in detail.^1^, ^2^, ^3^, ^4^ Differences in neural architecture and electrical signalling, ease of surgical access and implantation and physical dimensions dictate that the challenges inherent in stimulating at each anatomical target are diverse and complex.^1^

Regardless of the targeted location, the typical response to electrical stimulation is the eliciting of light percepts called *phosphenes* which, if sufficiently discrete in character and elicited in suitable numbers, may be utilized to provide a blind person with useful information about their surroundings. It is this fundamental observation that underpins the current efforts to develop visual prostheses.

At present, there are a large number of research groups internationally working towards the development of visual prostheses, covering each viable target region within the visual pathway. There are almost 20 research teams developing retinal prostheses with varying implant locations, with groups based in Australia, USA, Germany, France and Japan reporting progress in translating retinal visual prostheses from the experimental to clinical domains. The Argus II device (Second Sight Medical Products, Inc., Sylmar, CA, USA)^3^ and the Alpha IMS (Retina Implant AG, Reutlingen, Germany)^5^ received regulatory approval for clinical use in the European Union in 2011 and 2013 respectively, whilst the Argus II was approved for use by the FDA in 2013 under the Humanitarian Use Device programme. Clinical studies of these devices are demonstrating improvements in visual acuity and/or the ability to undertake activities of daily living.^6^, ^7^

Two groups have reported on the development of bionic vision devices based around electrodes implanted into or around the optic nerve^8^, ^9^ and another two have described the implantation of electrodes into the lateral geniculate nucleus of non‐human primates^10^ and rats,^11^ with the goal of developing a visual prosthesis based on stimulation of these targets.

Despite significant progress having been made since the first attempts to develop a cortical visual prosthesis over 50 years ago, 12 regulatory approval for these devices is yet to be granted. To our knowledge four groups (Australia, USA, Canada and Spain) are developing cortical visual prostheses,^1^ and clinical trials of these are anticipated within the next several years.

## Retinal and cortical prostheses: general considerations

### System architecture

There is some commonality in the architecture of electrically stimulating visual prostheses, whether retinal or cortical.^1^, ^2^, ^6^, ^7^, ^13^, ^14^ These common features include capture of digital images by a camera, typically built into a pair of glasses. Simplification of images and/or feature extraction will highlight objects, floor areas or printed text or simply enhance contrast prior to the generation of patterned electrical impulses for transmission to the electrode arrays. This transmission may take place across a wired or wireless connection, which may also be used to transfer power to the implanted electronics. The circuitry required to provide stimulus pulses to the electrodes can either be incorporated into the electrode array/s themselves or contained within a separate implanted package tethered to the electrodes.

A variation on this approach involves a light‐sensitive element (e.g. photodiodes) incorporated into the implant, wherein it may be combined with a stimulating electrode array directly into a single construct.^6^, ^15^ In one embodiment of this technique, image data is transmitted to the combined photodiode/electrode array by a miniature glasses‐mounted infrared projector, the light from which is also converted to electrical energy to power the electrodes.^15^ Another approach is to amplify weak photoelectric signals generated by ambient light incident on a photodiode array.^6^ This requires that the array be powered separately, which can be achieved by a wireless radiofrequency link.^6^ The latter method has the advantage of allowing for natural gaze fixation and eliminates the need for head movements when scanning the environment. Intraocular image capture has also been described for a retinal prosthesis; however, in principle, it could be extended to cortical devices.^1^

### Electrode implantation and stimulation

Of all known stimulation sites, the retina and visual cortex have the longest history of development. A prototype retinal stimulator was developed by Tassicker in 1956,^16^ whilst the first experimental cortical device was reported by Button in 1958. 12

When stimulating the retina, visual percepts are elicited via direct stimulation of surviving inner retinal cells (bipolar and ganglion cells). Axons from the ganglion cells converge at the optic disc to form the optic nerve, which conveys visual information distally. An advantage of the retinal approach is the ability to use the vision processing abilities of the inner retina to optimize the visual percepts. However, the requirement for viable inner retinal neurons renders retinal implants unsuitable for a number of blinding conditions, including glaucoma where these cells are lost, and traumatic ocular injury where the integrity of the globe is destroyed. To date, the majority of retinal prostheses have been implanted in patients with RP.

Electrical stimulation of the visual cortex may be achieved via surface or penetrating electrodes. From the perspective of eliciting smaller central phosphenes and thus providing higher acuity central vision, primary visual cortex (V1) offers some technical advantages. Retinocortical magnification, which results in a significant over‐representation of the foveal visual field on visual cortex, provides for a substantially greater surface area within which to implant stimulating electrodes and thus elicit central phosphenes.^17^ On the other hand, much of V1 is relatively inaccessible to the implantation of penetrating electrodes, and current cortical prosthesis development efforts are typically focused on devices that stimulate the occipital pole and its surroundings.^18^

Whilst surgical access to the occipital pole is relatively straightforward, neurosurgery undertaken for non‐life‐saving reasons is a prospect that requires careful planning from an ethical, medico‐legal and procedural perspective.^1^ Clearly, the implantation of retinal or suprachoroidal arrays carries a correspondingly lower risk of mortality and morbidity. Therefore, uncertainties about risk versus benefit must be addressed before implantations of cortical electrode arrays in blind individuals will pass regulatory scrutiny. In this context, psychophysical studies including simulated phosphene vision experiments will be of paramount importance in determining the likely functional benefit of prosthetic vision. Moreover, detailed preoperative assessment of the psychological fitness of potential implant recipients will be a key factor in maximizing the efficacy of post‐implantation visual rehabilitation and training. 1

A key challenge common to both retinal and cortical implants is maximizing central phosphene resolution and thus visual acuity, via the implantation of small, densely packed electrodes in either the macula in the retina, or occipital pole of the cerebral cortex. Limitations to this density arise due to a combination of factors, including but not limited to current spread from nearby electrodes causing phosphene fusion, and the interplay between the delivered charge, activation thresholds, electrode surface area and the likelihood of tissue or electrode damage.^19^ Beyond the limits of electrode size and array density imposed by physical and biological factors, further improvements in acuity will only be achievable with more sophisticated stimulation strategies, which continue to be developed.^19^

## Retinal visual prosthesis implantations and outcomes

**Figure 1:**
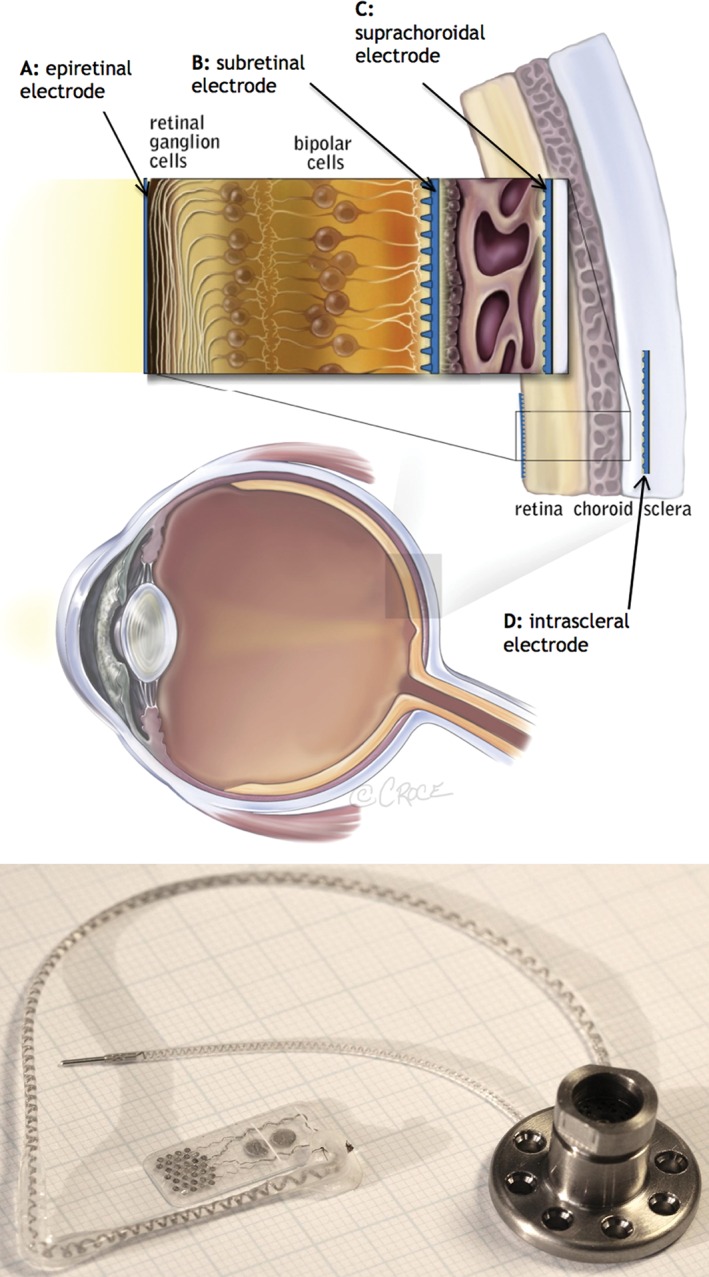
Top: Schematic representation of the human eye showing the surgical locations for epiretinal, subretinal, suprachoroidal and intrascleral electrodes. Reproduced from Ayton et al.,^14^ with permission under the terms of the Creative Commons Attribution License. Bottom: The prototype suprachoroidal implant used in the Bionic Vision Australia pilot study. The electrode array is composed of 33 platinum electrodes on a silicone substrate, of which 24 were able to be stimulated. The array was attached to a percutaneous connector via a helical lead wire, which was implanted behind the ear and allowed direct stimulation of the array. Image provided by Dr David Nayagam, Bionics Institute.

Of the various vision prosthesis modalities, retinal implants have had the highest number of human clinical trials to date. Bionic Vision Australia recently completed a clinical trial of a suprachoroidal implant, with the recipients (*n* = 3) showing sustained phosphene perception, stable implant location and significantly improved light location detection over a period of 12 months.^14^ Other clinical studies have shown efficacy for retinal prostheses placed in the epiretinal, subretinal and intrascleral locations (Fig. 1, top).^5^, ^20^, ^21^

All reported outcomes to date have been in patients with the end‐stage retinal degenerative diseases RP and choroideremia. In early 2015, surgeons in Manchester implanted the first epiretinal implant (Argus II) in patients with AMD, and pilot clinical results are anticipated in the near future. This important study looks to expand the number of patients who benefit from visual prostheses, but in doing so presents different challenges. In particular, patients with AMD have at risk their central vision only, with good function remaining in their peripheral vision. Thus, the implant must offer a greater level of visual acuity and functional benefit than is currently possible to justify the surgical procedure and potential risk to peripheral vision.

Surgical methods for the various retinal implant designs vary in complexity, and intraoperative and postoperative adverse events have been noted. These have included conjunctival and scleral erosions, retinal detachment, hypotony and endophthalmitis with epiretinal devices,^22^ increased retinal microaneurysms with subretinal devices^23^ and subretinal haemorrhage with suprachoroidal devices.^14^ From preliminary reports, it appears that suprachoroidal and intrascleral implantations offer improved surgical safety and stability, with fewer intraoperative complications.^14^, ^21^ This is likely due to the fact that the device does not require implantation through penetrating incisions into the posterior globe, nor mechanical attachment to the retinal surface.^14^ However, as the electrodes in these devices are further from the ganglion cells, patient perceptual thresholds for electrical stimulation are higher than in epiretinal or subretinal prostheses.^24^ Despite this, suprachoroidal and intrascleral electrode stimulation has proven effective.^20^, ^21^

Participants in retinal prosthesis trials to date have all had very little baseline vision, with most having ‘bare light perception’ only. From this profound impairment, retinal implant recipients have shown improvements in visual acuity, as measured using grating acuity tasks or Landolt C optotypes.^14^, ^25^, ^26^ All of these postoperative visual acuities classify as ‘ultra‐low vision’, and would not be sufficient to enable reading of standard size print or facial recognition. However, it has been shown that this level of vision can enable some patients to recognize large letters.^20^ Notably, camera functions (e.g. zoom) can improve visual acuity measures in retinal implants, albeit at the expense of a reduced visual field. For applications such as navigation, the visual field size and the apparent location of phosphenes are also important. It is desirable that patients can navigate around large objects without excessive head or eye scanning that results from narrow visual fields. If phosphenes are displaced relative to the straight‐ahead position, strategies such as adjusting head position or eye gaze direction may be required in order for objects to be perceived in the correct position.

Despite the variation in acuity measures from different implant designs, functional vision outcomes (which reflect how people use their prosthetic vision on real world tasks) have been relatively similar. Devices tested to date have enabled improvement on subjects’ ability to recognize objects on a table,^25^, ^26^ and to navigate around high‐contrast obstacles in orientation and mobility tasks.^25^ Such increases in independent mobility are the main goal for the present‐generation vision prostheses, and will remain a key element of vision restoration assessments in the future.^27^

## Cortical visual prosthesis implantations and outcomes

There is little contemporary data describing the stimulation of visual cortex electrode arrays in humans; the limited short‐ and long‐term data that is available principally derives from the work of Brindley and Dobelle. Brindley developed and implanted three prototype wirelessly operated electrode arrays, each of which successfully elicited large numbers of phosphenes.^28^ The arrays of platinum electrodes were embedded in silicon, moulded to fit the occipital poles of a ‘typical human brain’ plaster model.^29^ Dobelle developed a Teflon array which was inserted subdurally over the medial occipital cortex and connected to the stimulating electronics via a transcutaneous connector.^30^ The limitations of Dobelle's design are inherent in later reports of system failures, infections and seizures,^31^ suggesting that major modifications and/or improvements to such devices would be required before they could be considered fit for further clinical trials.

Limited human studies on intracortical microelectrodes were performed at the US National Institutes of Health in the 1990s, which demonstrated their ability to deliver highly focal stimuli at low stimulus currents.^32^ These experiments demonstrated that intracortical microstimulation could elicit phosphenes in a blind subject who also reported no visual percepts from cortical surface stimulation. Six electrodes were concurrently stimulated; however the contemporary belief is that a functional prosthesis should elicit many more phosphenes to provide measurable improvements in mobility, object recognition and the reading of printed text.^33^ Notably, however, a recent survey conducted with recipients of the Dobelle implants suggests that the functional benefits were obtained across the entire spectrum of phosphene map sizes, ranging from 119 phosphenes to just seven.^34^ Thus, there may be substantial variability in the experiences of individual prosthesis recipients, independent of the number of phosphenes reported. Carefully designed training and rehabilitation programmes will therefore be necessary to achieving successful outcomes,^35^ as will the development of appropriate tools for quantifying that success.^27^, ^36^

**Figure 2:**
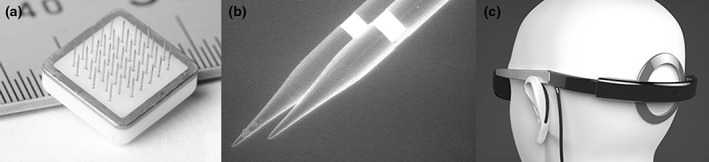
(a) A close‐up view of a single cortical visual prosthesis tile with 43 penetrating electrodes, as developed by the Monash Vision Group. (b) Scanning electron micrograph of the electrodes, showing an annular stimulating surface approximately 500 μm from the tips. (c) Artist's rendering of the headwear, showing the data and power transmitting/receiving coil overlying the recipient's occiput and the implanted tiles. (a and b) Reproduced from Lowery et al.,^18^ with permission (© 2015 IEEE). (c) Supplied courtesy of Monash Art, Design and Architecture and Monash Vision Group.

Current efforts to develop a cortical visual prosthesis remain centred around the concept of high‐density stimulation via arrays of intracortical microelectrodes (Fig. 2) implanted into the occipital pole or its immediate surroundings.^37^, ^38^ This approach takes advantage of the high cortical magnification at this location to provide a theoretically denser central phosphene map.

Clinical experience with the experimental implantation of cortical penetrating electrode arrays is largely limited to temporary implantations in patients undergoing epilepsy or brain tumour surgery,^4^ or to patients participating in longer term trials of experimental cortical motor prostheses.^39^, ^40^ For the latter, the available information suggests most patients experience an uneventful recovery from implantation surgery.

Beyond the initial electrode implantations and a successful recovery from surgery, numerous challenges will need to be overcome in order to achieve a functional cortical visual prosthesis in the long term.^1^, ^4^, ^41^, ^42^

## Novel approaches

Beyond the direct injection of electrical current using electrodes, artificial stimulation of neural pathways can also be achieved using alternating magnetic fields,^43^ low‐intensity focused ultrasound,^44^ optogenetics,^45^ thermal changes and using microfluidic devices to inject neurotransmitters or to alter ionic gradients across neural cell membranes.^46^

Aside from novel techniques for stimulation itself, new methods for interfacing neural tissue with conventional electrodes are being developed. A group based in Japan has cultured neurons directly onto a microelectromechanical substrate, which then interface to the central nervous system neurons (e.g. retinal ganglion cells) by growing along nerve guides. Using such techniques, it is anticipated that more reliable connections between the electrodes and the target neurons may be achievable.^47^

## Outlook and future directions

The outlook for prosthetic vision devices is largely dependent on achieving the dual goals of validating safe techniques for surgically implanting electrode arrays,^48^, ^49^ and the necessary demonstration of clinical efficacy that must precede regulatory approval.^22^ This is a particularly pressing issue for cortical devices, given the need to perform a craniotomy to obtain access to the cortical surface. Safety is of paramount importance; with safe electrode implantation techniques in place, it will be possible to continue developing improved stimulation strategies,^24^, ^50^ image processing algorithms^51^ and psychophysical assessment paradigms^27^, ^36^ to optimize efficacy. Further improvements may result in these devices becoming viable vision restoration options for a broader range of blinding conditions, and even for those with some residual vision.

Whilst great progress has been made in the development of visual prostheses, research into alternative therapies and/or vision restoration strategies continues apace. For example, subretinal injections of human embryonic stem cells have demonstrated safety and vision improvement in patients with AMD and macular dystrophy.^52^ Gene transfer therapies have been trialled in patients with Leber's congenital amaurosis,^53^ with trial subjects showing sustained vision improvements over a 2‐year period.^54^ Optogenetic or optopharmacological methods may be used to confer light sensitivity to retinal ganglion cells in RP and AMD,^55^ and neurotrophic factors may be administered intravitreally to support and/or regenerate retinal neurons affected by glaucoma.^56^

Interestingly, retinal stimulation has been found to have a neurotrophic, anti‐apoptotic and anti‐inflammatory effect,^57^, ^58^ delaying photoreceptor degeneration in rat models of RP and preserving the function of retinal ganglion cells after experimental injury.^59^ This effect was hypothesized in recipients of the artificial silicon retina subretinal implant, who reported improvements in baseline visual function with the device switched off, between 1 week and 2 months after surgery.^60^

In summary, electrical stimulation of the visual pathways is a viable strategy for vision rehabilitation in the blind, and clinical translation of devices that stimulate the retina is underway. Several cortical devices are being developed, for which first‐in‐human trials may be conducted in the near future. Multiple biological therapies are being explored in parallel, and it remains to be seen how these competing or complementary approaches will compare, with regards to their safety, longevity, and the degree of improvement in functional vision that can be achieved.
